# From support and interaction to continuance: affective pathways and the bright side of cognitive load in English-medium instruction

**DOI:** 10.3389/fpsyg.2026.1806932

**Published:** 2026-07-22

**Authors:** Wenyan Yi

**Affiliations:** School of International Communication, Wuhan Business University, Wuhan, China

**Keywords:** cognitive load, continuance intention, English-medium instruction, learning enjoyment, learning satisfaction, perceived interaction, perceived support

## Abstract

English-medium instruction (EMI) is increasingly adopted in higher education, yet learners differ in their willingness to persist in EMI courses. While prior research has highlighted the cognitive and emotional demands of EMI learning, the relationships among these demands, learners’ affective evaluations, and their continuance intention remain insufficiently examined. Guided primarily by expectation-confirmation logic and supplemented by positive emotion and cognitive load perspectives, this study examines an affective-cognitive model linking perceived support, perceived interaction, learning enjoyment, learning satisfaction, cognitive load, and continuance intention in EMI learning. Survey data were collected from 320 university students enrolled in EMI courses, and the proposed model was tested using structural equation modeling. The findings indicate that perceived support and perceived interaction are associated with distinct affective pathways. Perceived support is directly associated with learning satisfaction, reflecting learners’ evaluative judgments of their overall learning experience, whereas perceived interaction is primarily related to learning enjoyment as an affective response to interactive EMI learning experiences. Learning enjoyment is positively associated with learning satisfaction, which emerges as the most proximal predictor of continuance intention. In addition, cognitive load positively moderates the relationship between learning enjoyment and learning satisfaction, indicating that enjoyment is more strongly related to satisfaction when learners report higher cognitive load. By conceptualizing learning satisfaction as an affective evaluation mechanism rather than a terminal outcome, this study clarifies the structural role of satisfaction in continuance intention in EMI learning. The findings suggest that, rather than simply reducing difficulty, effective EMI design should align instructional support and interaction with learners’ affective evaluations in cognitively demanding EMI contexts.

## 1 Introduction

Over the past two decades, English-medium instruction (EMI) has become an increasingly prominent feature of higher education worldwide, especially in non-Anglophone settings where universities pursue internationalization and global competitiveness (Macaro et al., 2018; Pun and Jin, 2022). It is commonly presented as a strategic tool for strengthening students’ academic and linguistic resources while also enhancing institutions’ international visibility and appeal (Soruç et al., 2024; Wang, 2026). Yet, empirical research has consistently shown that students’ experiences of EMI vary widely. Across EMI learning contexts, learners report markedly different levels of satisfaction, emotional engagement, and willingness to continue learning through EMI (Dewaele and MacIntyre, 2014; Hu and Wu, 2020). While many students acknowledge the instrumental value of EMI, they also describe emotional strain, heightened anxiety, and doubts about whether sustained engagement in EMI learning is worth the effort (Dewaele and MacIntyre, 2014; Xu et al., 2025; Peng and Zhou, 2025). Taken together, these mixed accounts suggest that EMI learning is shaped not only by cognitive demands but also by learners’ emotional responses, raising a central question concerning the relationships among demanding EMI experiences, learners’ affective evaluations, and their willingness to persist.

Existing EMI research has approached variation in learners’ satisfaction and willingness to persist through several complementary perspectives. One influential perspective foregrounds learners’ English language proficiency, suggesting that limited linguistic resources heighten cognitive strain and constrain learners’ ability to engage productively with EMI learning (Macaro et al., 2018; Pun and Jin, 2022). A second line of inquiry shifts attention toward instructional and contextual conditions, including perceived teacher support, classroom interaction, and pedagogical design, which shape the quality of learners’ EMI experiences (Hu and Wu, 2020). Drawing on cognitive load theory, subsequent studies have increasingly characterized EMI as a learning mode that places substantial demands on cognitive processing, often treating cognitive load as a limiting factor that impedes comprehension and learning efficiency, following foundational formulations by Sweller (1988) and Paas et al. (2003). Taken together, this body of work has clarified the linguistic, instructional, and cognitive conditions under which learners are more likely to engage successfully in EMI learning. What remains less clearly articulated is the relationship between demanding EMI experiences, learners’ affective evaluations, and their intention to continue EMI learning.

Two closely connected gaps remain insufficiently addressed in the EMI literature. Although learning satisfaction is frequently examined in EMI research, it is seldom conceptualized as an affective evaluation mechanism that actively shapes learners’ decisions about continued engagement. More often, satisfaction is positioned as a distal outcome, rather than as an evaluative mechanism linking learning experiences to persistence-related judgments (Bhattacherjee, 2001; Hu and Wu, 2020). At the same time, continuance intention—despite its central role in learning systems and user behavior research—has received limited attention in EMI contexts, which tend to prioritize achievement outcomes and momentary emotional responses over longer-term engagement decisions (Bhattacherjee, 2001; Ye et al., 2022; Zhang et al., 2023; Alterkait and Alduaij, 2024). Beyond these gaps, prior EMI and related online-learning research has often emphasized the potential costs of high cognitive demand, including increased processing difficulty and weaker evaluative outcomes (Macaro et al., 2018; Pun and Jin, 2022; Cheng et al., 2023). This emphasis leaves less room for examining whether cognitive effort in demanding learning environments may also carry different evaluative meanings when it remains manageable (Skulmowski and Xu, 2022; Zeitlhofer et al., 2024). Consequently, the relationships between cognitive demands, learners’ affective appraisal of EMI experiences, and the evaluative significance of positive emotions in challenging EMI contexts require further clarification.

In response to these unresolved issues, this research is guided primarily by expectation-confirmation logic and supplemented by positive emotion and cognitive load perspectives. Within this framework, learning satisfaction is conceptualized as an affective evaluation mechanism rather than a terminal outcome, linking learners’ EMI experiences with their continuance intention. By bringing together learners’ EMI experiences, affective appraisal processes, and cognitive demands within a single explanatory framework, this research contributes to the EMI literature in several respects. At a theoretical level, it extends satisfaction-continuance frameworks to cognitively demanding multilingual learning contexts by linking learners’ affective evaluations and cognitive demands with continuance intention. From a practical perspective, the findings underscore the need to design EMI environments in which instructional support and interaction are aligned with learners’ affective evaluations, redirecting attention from simply lowering difficulty toward cultivating conditions that support learners’ willingness to persist in the face of challenge.

## 2 Theoretical background and hypotheses development

### 2.1 EMI learning as a cognitively and affectively demanding context

English-medium instruction (EMI) has commonly been examined as a learning context associated with heightened cognitive demands, particularly within non-Anglophone higher education systems (Long et al., 2025; Wang, 2026). In contrast to first-language instruction, EMI requires learners to engage with disciplinary knowledge through a second language, necessitating the concurrent processing of subject content and linguistic form. Cognitive load theory suggests that this dual-processing requirement places considerable demands on working memory and attentional resources, frequently leading to increased cognitive effort and reduced processing efficiency (Sweller, 1988; Paas et al., 2003; Macaro et al., 2018; Pun and Jin, 2022).

Alongside these cognitive challenges, an expanding body of research has drawn attention to the affective complexity of EMI learning. Empirical studies consistently show that EMI learners experience heightened anxiety and emotional uncertainty, often rooted in linguistic insecurity and concerns about negative evaluation (Dewaele and MacIntyre, 2014; Dai and Wang, 2024). At the same time, EMI learning cannot be reduced to a purely negative emotional experience. When learners perceive themselves as capable of managing linguistic and conceptual demands, EMI classrooms may also evoke positive affective experiences, including enjoyment, a sense of challenge, and academic accomplishment (Xu et al., 2025). Taken together, these findings indicate that EMI learning is best understood as an emotionally ambivalent process in which strain and engagement coexist (Hillman et al., 2023; Sun and Yang, 2025).

Despite this recognition, much of the EMI literature continues to treat cognitive and affective dimensions as parallel yet largely independent strands. Cognitive load is typically conceptualized as a constraint that impedes comprehension and learning outcomes (Sweller, 1988; Paas et al., 2003), whereas affective variables—such as anxiety or enjoyment—are often examined as discrete emotional outcomes rather than as integral components of learners’ meaning-making processes (Dewaele and MacIntyre, 2014). As a result, these approaches risk oversimplifying EMI learning by overlooking the affective evaluation of cognitively demanding experiences.

More critically, limited attention has been paid to the relationship among cognitively and affectively demanding EMI experiences, evaluative judgments, and longer-term learning decisions. Although recent studies have documented learners’ emotional experiences in EMI classrooms, including enjoyment and anxiety (Dai and Wang, 2024; Xu et al., 2025; Sun and Yang, 2025), the relationships among affective experiences, learning satisfaction, and persistence-related intentions under cognitively demanding EMI conditions remain insufficiently theorized.

Recent research suggests that cognitive load should not be understood solely as a detrimental burden (Skulmowski and Xu, 2022; Zeitlhofer et al., 2024). In highly demanding learning environments such as EMI, sustained cognitive effort may also reflect learners’ active engagement and meaningful investment in learning tasks (Pun and Jin, 2022). Learning enjoyment that emerges under higher levels of cognitive load is more likely to be interpreted as meaningful and worthwhile rather than superficial. In this sense, cognitive load may amplify the evaluative significance of positive emotional experiences, strengthening their influence on overall learning satisfaction.

These arguments point to the affective-cognitive model of EMI continuance proposed in this study. Expectation-confirmation logic provides the main organizing logic by treating learning satisfaction as an evaluative mechanism through which learners’ EMI experiences are linked to continuance intention. Within this logic, perceived support is conceptualized as an experiential resource that may contribute to satisfaction, whereas perceived interaction is expected to shape satisfaction indirectly by first eliciting learning enjoyment. Positive emotion research helps explain the affective pathway from perceived interaction to learning enjoyment and from learning enjoyment to satisfaction. Cognitive load theory further accounts for the boundary condition in this process, suggesting that the evaluative meaning of enjoyment may vary depending on the level of cognitive load experienced by learners. In this way, the model connects learning experiences, positive emotion, evaluative judgment, and cognitive demand in explaining EMI continuance.

### 2.2 Perceived support and learning satisfaction in EMI

Perceived support can be broadly understood as learners’ subjective assessment of the extent to which assistance is available and adequate throughout the learning process, including instructional, emotional, and informational support (Hu et al., 2023). In EMI contexts, perceived support is commonly manifested in teachers’ clarity of explanation, responsiveness to learners’ difficulties, and the use of scaffolding practices designed to reduce linguistic and conceptual barriers (Hu and Wu, 2020). Given the heightened cognitive and linguistic demands inherent in EMI learning, perceived support constitutes a critical experiential resource that shapes how learners interpret learning demands and regulate their engagement with challenging academic tasks.

Expectation confirmation theory (ECT) and its extensions conceptualize learning satisfaction as an evaluative judgment formed through learners’ comparisons between prior expectations and subsequent learning experiences (Bhattacherjee, 2001). In EMI learning, where learners must sustain substantial cognitive effort while also managing emotional pressure, perceived support assumes particular importance in shaping these evaluative processes. In supportive EMI learning environments, reduced uncertainty and perceived risk allow learners to appraise EMI learning as manageable, worthwhile, and valuable rather than overwhelming or discouraging.

Prior EMI research has emphasized the importance of instructional and contextual support for learners’ EMI experiences (Hu and Wu, 2020), while related e-learning research has linked learning-environment quality to student satisfaction (Alterkait and Alduaij, 2024). However, existing research has devoted limited attention to the affective evaluation processes through which support operates under cognitively demanding EMI conditions. As a result, perceived support has rarely been examined as part of a broader affective pathway linking supportive experiences to persistence-related outcomes.

Against this backdrop, perceived support is conceptualized in the present study as an experiential input that contributes to learning satisfaction by shaping learners’ evaluative interpretations of demanding EMI learning experiences. Accordingly, learning satisfaction functions as an affective evaluation mechanism linking supported learning experiences with judgments about overall learning quality in EMI contexts.

*H1*: Perceived support is positively associated with learning satisfaction in EMI learning.

### 2.3 Perceived interaction and learning enjoyment in EMI

Perceived interaction can be broadly understood as learners’ subjective perceptions of the extent and quality of their exchanges with instructors and peers during learning activities (Pan et al., 2024). In EMI classrooms, interaction plays a central role in supporting meaning negotiation, fostering social presence, and sustaining engagement under linguistically and cognitively demanding conditions (Kırkgöz et al., 2023). From learners’ perspectives, interactional experiences offer opportunities not only for academic clarification but also for emotional affirmation and communicative validation.

Meaningful interaction allows cognitively demanding learning tasks to be experienced as engaging rather than burdensome. Prior EMI research indicates that high-quality interaction is closely associated with positive academic emotions—particularly learning enjoyment—as learners experience challenge alongside perceived competence, communicative success, and social support (Dai and Wang, 2024). Under these conditions, interaction functions as a source of emotional resonance that helps learners interpret effortful engagement as enjoyable rather than aversive.

Despite these insights, EMI research has often examined interaction primarily in relation to linguistic performance, participation patterns, or discourse practices, while paying comparatively limited attention to its role in shaping learners’ positive academic emotions. In particular, although interaction is widely recognized as an important feature of EMI classrooms, its role as a proximal antecedent of learning enjoyment—rather than as a general contextual factor or a direct predictor of achievement outcomes—has received relatively limited explicit attention in existing EMI research.

Against this backdrop, perceived interaction is conceptualized as a key experiential antecedent of learning enjoyment in EMI learning, emphasizing its role in shaping learners’ affective pathways to cognitively demanding learning experiences.

*H2*: Perceived interaction is positively associated with learning enjoyment in EMI learning.

### 2.4 Learning enjoyment and learning satisfaction in EMI

Learning enjoyment is a positive activating emotion that emerges when learners engage with challenging yet meaningful learning activities and experience a sense of competence and value in the learning process (Pekrun, 2006; Li and Xing, 2024; Pekrun, 2024). Related EFL research on flow has also treated enjoyment as an important dimension of positive English learning experience within a broader multidimensional structure of learning flow (Jia et al., 2025). In EMI learning, enjoyment typically reflects learners’ successful management of linguistic and cognitive challenges rather than the simplicity of learning tasks. When enjoyment arises under such conditions, it signals that learners interpret effortful engagement as worthwhile, thereby shaping subsequent evaluative judgments of their learning experiences.

More broadly, positive emotions such as enjoyment play a critical role in shaping evaluative outcomes. According to the broaden-and-build theory of positive emotions, enjoyment expands learners’ cognitive and motivational resources, facilitating flexible thinking, meaning-making, and positive appraisal of ongoing experiences (Fredrickson, 2001). In EMI contexts, learning enjoyment may enhance perceived value, alleviate emotional strain, and support more favorable evaluations of learning experiences, thereby contributing to higher levels of learning satisfaction.

Despite increasing attention to enjoyment in EMI classrooms, existing research has often treated enjoyment primarily as an affective outcome of instructional conditions rather than as an affective input that informs evaluative judgments. As a result, the role of learning enjoyment in the formation of learning satisfaction—particularly under cognitively demanding EMI conditions—remains insufficiently theorized.

Against this backdrop, learning enjoyment is conceptualized as a key affective driver of learning satisfaction in EMI learning, emphasizing its role in shaping learners’ evaluative interpretations of effortful and demanding learning experiences.

*H3*: Learning enjoyment is positively associated with learning satisfaction in EMI learning.

### 2.5 Learning satisfaction and continuance intention in EMI

Learning satisfaction represents learners’ overall affective evaluation of their learning experiences and occupies a central position within expectation confirmation theory (ECT) and its derivative models (Oliver, 1980; Bhattacherjee, 2001). Rather than functioning only as a distal outcome, satisfaction can be treated as an evaluative link between prior learning experiences and continuance intention (Bhattacherjee, 2001).

In EMI learning, persistence entails sustained cognitive investment, ongoing negotiation of linguistic and academic demands, and continuous emotional regulation (Macaro et al., 2018; Pun and Jin, 2022; Peng and Zhou, 2025). Consistent with continuance research, continuance intention reflects an evaluative judgment shaped by prior learning experiences and satisfaction rather than an automatic continuation of learning activities (Bhattacherjee, 2001; Ye et al., 2022; Zhang et al., 2023). Learners are therefore unlikely to persist unless their accumulated learning experiences are appraised as sufficiently valuable and satisfying. Learning satisfaction thus plays a particularly critical role in shaping continuance intention in EMI contexts, as it encapsulates learners’ judgments about whether the benefits of EMI learning justify its cognitive and emotional costs.

Prior research in information systems and online learning contexts has consistently identified satisfaction as a central antecedent of continuance intention, underscoring its role in persistence-related decision-making (Bhattacherjee, 2001; Ye et al., 2022; Zhang et al., 2023). Despite this theoretical relevance, continuance intention remains underexplored in EMI research, which has predominantly focused on achievement outcomes or immediate affective pathways. This gap underscores the need to examine satisfaction-driven persistence decisions within EMI learning environments.

On this basis, learning satisfaction is expected to play a central role in shaping learners’ continuance intention in EMI learning.

*H4*: Learning satisfaction is positively associated with continuance intention in EMI learning.

### 2.6 Mediating roles of learning enjoyment and learning satisfaction in EMI

In EMI learning, learners’ continuance intention is unlikely to emerge directly from isolated learning experiences. Rather, persistence-related judgments may be shaped through indirect affective pathways, in which learners’ emotional responses to demanding learning experiences are incorporated into broader evaluations of learning quality. This argument is relevant in EMI contexts, where continued participation involves considerable cognitive effort, linguistic demands, and emotional regulation (Macaro et al., 2018; Pun and Jin, 2022; Peng and Zhou, 2025). It is also consistent with continuance and emotion research, which highlights the role of satisfaction in shaping continuance intention and the role of positive emotions in broadening learners’ evaluative and motivational resources (Bhattacherjee, 2001; Fredrickson, 2001; Pekrun, 2006).

Within this framework, perceived interaction is expected to influence learning satisfaction indirectly through the generation of learning enjoyment. Meaningful interactional experiences can elicit enjoyment by enabling communicative success, social connection, and a sense of competence under linguistically demanding conditions. When enjoyment arises under challenging EMI learning conditions, it enhances learners’ perceived value of engagement and contributes to more favorable evaluations of overall learning quality. Accordingly, learning enjoyment is expected to mediate the relationship between perceived interaction and learning satisfaction.

*H5*: In EMI learning, learning enjoyment mediates the relationship between perceived interaction and learning satisfaction.

In a similar vein, perceived support is not expected to translate automatically into continuance intention. Instead, supportive learning experiences shape persistence-related decisions by informing learners’ evaluative judgments of learning satisfaction. In EMI contexts, where continued participation requires sustained investment, learners are unlikely to maintain engagement unless their accumulated learning experiences are appraised as sufficiently supportive and worthwhile. Learning satisfaction therefore functions as a key evaluative mechanism through which perceived support influences continuance intention.

*H6*: In EMI learning, learning satisfaction mediates the relationship between perceived support and continuance intention.

Finally, learning enjoyment is expected to contribute to continuance intention indirectly through its influence on learning satisfaction. As a positive activating emotion, enjoyment signals that effortful engagement is meaningful and rewarding; however, such emotional experiences must be integrated into broader evaluative judgments before they can inform persistence decisions. Learning satisfaction thus serves as an evaluative bridge linking enjoyment with continuance intention in EMI learning.

*H7*: In EMI learning, learning satisfaction mediates the relationship between learning enjoyment and continuance intention.

### 2.7 Moderating role of cognitive load in EMI

Cognitive load theory has traditionally conceptualized cognitive load as a limiting factor that constrains learning when processing demands exceed learners’ cognitive capacity (Sweller, 1988). Accordingly, excessive cognitive load is expected to undermine comprehension and learning effectiveness. At the same time, cognitive load does not necessarily indicate overload across all learning situations (Skulmowski and Xu, 2022). In cognitively demanding contexts such as EMI learning, cognitive load may also reflect learners’ active cognitive investment in engaging with complex linguistic and disciplinary content, provided that such demands remain within manageable limits.

When learning enjoyment is experienced under conditions of relatively high but manageable cognitive load in EMI learning, it signals sustained engagement with challenging tasks rather than superficial participation. Under such circumstances, enjoyment is more likely to be interpreted as evidence that effortful engagement is meaningful and worthwhile, thereby exerting a stronger influence on learners’ evaluative judgments of learning experiences. Cognitive load therefore functions as a boundary condition that shapes the strength of the relationship between learning enjoyment and learning satisfaction in EMI contexts.

Against this backdrop, cognitive load is conceptualized as a boundary-condition moderator that strengthens the relationship between learning enjoyment and learning satisfaction under demanding but manageable learning conditions.

*H8*: In EMI learning, cognitive load positively moderates the relationship between learning enjoyment and learning satisfaction, such that the positive effect of learning enjoyment on learning satisfaction is stronger when cognitive load is relatively high.

### 2.8 Research model and hypotheses summary

Building on the preceding theoretical review, this study advances an integrative research model that examines the relationships among EMI learning experiences, affective pathways, cognitive load, and continuance intention. The model conceptualizes EMI learning as a cognitively and affectively demanding context in which learners form evaluative judgments based on perceived support, perceived interaction, and emotional engagement.

Within the proposed model, perceived support and perceived interaction are positioned as two core experiential inputs in EMI learning, operating through distinct yet complementary affective pathways. Perceived support is expected to influence learning satisfaction by shaping learners’ evaluative interpretations of demanding learning experiences. By contrast, perceived interaction is proposed to primarily foster learning enjoyment by enhancing emotional engagement, perceived competence, and meaning in learning activities. Learning enjoyment, in turn, contributes to learning satisfaction, highlighting the role of positive activating emotions in shaping evaluative judgments under conditions of challenge.

Learning satisfaction occupies a pivotal position in the model as an affective evaluation mechanism linking learning experiences and emotional responses to persistence-related decisions. Rather than being treated merely as an outcome, satisfaction is conceptualized as an affective evaluation mechanism linking cognitively and affectively demanding EMI experiences with judgments about continued engagement.

In addition, the model incorporates cognitive load as a moderating condition that shapes the strength of the affective pathway between learning enjoyment and learning satisfaction. Cognitive load is conceptualized as an indicator of cognitive investment that, under demanding but manageable learning conditions, amplifies the evaluative significance of learning enjoyment. When enjoyment is experienced alongside higher levels of cognitive load, its positive effect on learning satisfaction is expected to be strengthened.

**Figure 1:**
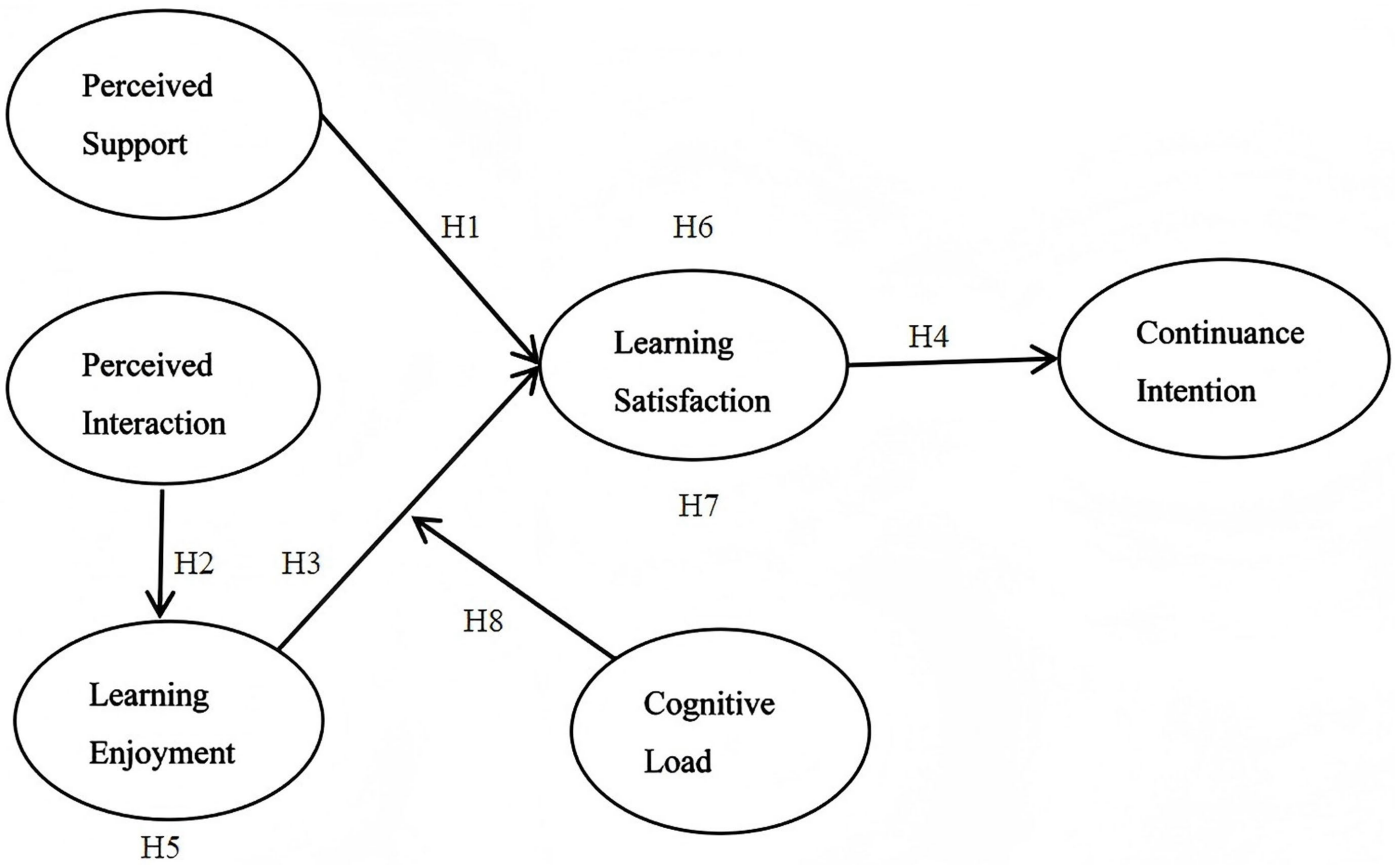
The conceptual model.

Taken together, the proposed research model specifies a set of direct, mediating, and moderating relationships (H1–H8) among learning experiences, affective evaluations, cognitive load, and continuance intention in EMI learning. Figure 1 presents the conceptual model and the hypothesized relationships examined in this study.

## 3 Research methodology

### 3.1 Variable measurement and questionnaire design

The questionnaire was developed by adapting multi-item scales from previous studies. As these scales were originally designed for related learning contexts rather than EMI specifically, each item was reviewed and adapted to fit the present study. Items were retained when their content matched the six constructs examined in the model: perceived support, perceived interaction, learning enjoyment, learning satisfaction, continuance intention, and cognitive load. The main adaptation involved revising the contextual wording of the items while keeping their original conceptual meanings. For example, references to general online learning, platform use, course learning, or broad learning experiences were reworded as “EMI course learning,” “EMI courses,” or “learning through EMI courses” when needed. During the adaptation and translation process, the retained items were translated into Chinese and adjusted to fit the EMI context. A back-translation into English was then conducted by a teacher with expertise in translation. The Chinese version and the back-translated English version were compared item by item to check whether the items conveyed the intended meanings clearly in the EMI context. All items measuring the six latent constructs were rated on a seven-point Likert scale ranging from 1 (strongly disagree) to 7 (strongly agree). Two demographic questions were also included to collect information on gender and academic year, using categorical response options.

As a preliminary check of the suitability of the item correlation matrix for factor analysis, the Kaiser–Meyer–Olkin (KMO) value and Bartlett’s test of sphericity were examined. The KMO value was 0.963, and Bartlett’s test of sphericity was significant, *χ*^2^ = 7024.973, df = 325, *p* < 0.001, indicating that the correlation matrix was suitable for factor analysis. The psychometric properties of the measurement scales were further evaluated using construct-level Cronbach’s *α*, confirmatory factor analysis, composite reliability, AVE, HTMT, and chi-square difference tests, as reported below and in the measurement model assessment.

Perceived support was measured with four items adapted from Hu et al. (2023) and Cheng et al. (2023). These items focused on learners’ perceptions of instructional, emotional, and informational assistance in EMI learning. The items were worded to capture guidance from teachers and classmates, emotional support, informational advice, and practical help in EMI course learning. A sample item is: “When I encounter difficulties in EMI course learning, I can receive emotional support, such as empathy and care, from teachers and classmates.” Higher scores indicated higher levels of perceived support in EMI learning. In the present study, the perceived support scale showed acceptable internal consistency, with a Cronbach’s *α* of 0.852.

Perceived interaction was assessed with three items adapted from Cheng et al. (2023) and Pan et al. (2024). These items reflected the perceived extent and quality of learner–teacher and peer interaction in EMI courses. The wording was adapted to refer specifically to communication, feedback, and discussion during EMI course learning. A sample item is: “In EMI course learning, teachers can respond to my questions and provide feedback in a timely manner.” Higher scores indicated stronger perceived interaction in EMI learning. In the present study, the perceived interaction scale showed acceptable internal consistency, with a Cronbach’s *α* of 0.737.

Learning enjoyment was measured with five items adapted from Alam et al. (2021). These items captured learners’ positive emotional engagement during EMI learning. The original wording was adapted to focus on students’ enjoyment of EMI course learning rather than general learning experiences. A sample item is: “I enjoy the learning process of EMI courses.” Higher scores indicated higher levels of learning enjoyment in EMI learning. In the present study, the learning enjoyment scale showed good internal consistency, with a Cronbach’s *α* of 0.939.

Learning satisfaction was assessed with five items adapted from Du et al. (2023) and Pan et al. (2024). These items reflected learners’ overall evaluations of their EMI learning experiences, including satisfaction with the learning process, learning outcomes, and overall experience. The wording was revised to refer specifically to EMI courses. A sample item is: “Overall, I am satisfied with the learning process of EMI courses.” Higher scores indicated higher levels of learning satisfaction with EMI learning. In the present study, the learning satisfaction scale showed good internal consistency, with a Cronbach’s α of 0.924.

Continuance intention was measured with four items adapted from Taghizadeh et al. (2021) and Alam et al. (2021). These items focused on learners’ willingness to continue engaging in EMI learning. The wording was adapted to capture students’ willingness to continue learning through EMI courses in relation to their current learning experiences and future development needs. A sample item is: “Based on my current learning experience, I am willing to continue learning through EMI courses.” Higher scores indicated stronger continuance intention toward EMI learning. In the present study, the continuance intention scale showed good internal consistency, with a Cronbach’s α of 0.869.

Cognitive load was measured with five items adapted from Cheng et al. (2023). These items covered learners’ perceived frustration, pressure, effort, complexity, and difficulty during EMI learning. The wording was adapted to refer to students’ cognitive experiences when learning disciplinary content through English. A sample item is: “EMI course learning makes me feel very stressed.” Higher scores indicated higher levels of perceived cognitive load in EMI learning. In the present study, the cognitive load scale showed good internal consistency, with a Cronbach’s α of 0.927.

### 3.2 Data collection and sample profile

Empirical data were collected via Credamo, a professional online survey platform in China. Data collection was conducted during the first quarter of 2025. Participants were undergraduate students enrolled in higher education institutions who had prior experience with EMI courses.

Participation in the survey was entirely voluntary. Before completing the survey, participants were informed of the academic purpose of the study, assured of the anonymity of their responses, and provided informed consent electronically. No personally identifiable information was collected. To encourage participation, respondents received a modest platform-based monetary incentive in accordance with the platform’s standard policies.

A total of 394 questionnaires were returned. Prior to data analysis, all responses were screened for quality. Questionnaires were excluded if they met any of the following criteria: unusually short completion time, consistent straight-line response patterns across all main scale items, or abnormal account or IP information identified during platform screening. After applying these exclusion criteria, 320 valid questionnaires were retained for subsequent analysis, giving a valid response rate of 81.22%. The final sample consisted of 41.88% male and 58.12% female students. In terms of academic standing, first-year students accounted for 17.19% of the sample, second-year students for 25.00%, third-year students for 28.13%, and fourth-year students for 29.68%. Overall, the sample exhibited a relatively balanced distribution across gender and academic year, supporting its suitability for the subsequent analyses.

To further assess the adequacy of the sample size, an *a priori* power analysis was conducted using G*Power 3.1 (Faul et al., 2009; Memon et al., 2020). The calculation was specified as an *F* test for linear multiple regression with a fixed model and *R*^2^ deviation from zero, with four predictors included. Using a conservative small-to-medium effect size of *f*^2^ = 0.08, an alpha level of 0.05, and a desired statistical power of 0.95, the required minimum sample size was 237. The final sample of 320 exceeded this requirement, indicating that the sample size was sufficient for the present study.

### 3.3 Data analysis strategy

Data analysis was conducted using covariance-based structural equation modeling in AMOS. Given that the proposed model involved six latent constructs, this approach allowed the measurement model and the hypothesized direct, mediating, and moderating relationships to be examined within a single analytical framework.

To assess the measurement model, confirmatory factor analysis (CFA) was first conducted. CFA was appropriate because the constructs and their indicators were specified in advance based on prior theory and established scales. Standardized factor loadings, composite reliability (CR), average variance extracted (AVE), and discriminant validity were examined to assess the reliability and validity of the six constructs, following commonly used criteria for measurement model evaluation. Discriminant validity was assessed using HTMT values with bootstrapped confidence intervals and chi-square difference tests between pairs of constructs. Because the data were collected through a self-reported questionnaire, common method variance was also assessed using a single-factor CFA, with all measurement items specified to load on one common factor.

After the measurement model had been evaluated, the structural model was estimated to test the direct relationships proposed in H1–H4. These paths included perceived support to learning satisfaction, perceived interaction to learning enjoyment, learning enjoyment to learning satisfaction, and learning satisfaction to continuance intention. The structural model allowed these relationships among latent constructs to be examined simultaneously while accounting for measurement error.

The mediating effects proposed in H5–H7 were examined using a bootstrap procedure with 5,000 resamples. Given that indirect effects are products of path coefficients and often have non-normal sampling distributions, bootstrap confidence intervals were used to assess their significance (Preacher and Hayes, 2008). An indirect effect was considered significant when its 95% confidence interval did not include zero.

To test the moderating effect proposed in H8, the composite scores of learning enjoyment and cognitive load were mean-centered, and their interaction term was entered as a predictor of learning satisfaction. This analysis examined whether the relationship between learning enjoyment and learning satisfaction varied according to the level of cognitive load.

## 4 Results

### 4.1 Measurement model assessment

The measurement model showed acceptable reliability and validity. Model fit was evaluated using commonly reported SEM fit indices, including *χ*^2^/df, GFI, IFI, TLI, CFI, and SRMR (Hu and Bentler, 1999; Hair et al., 2009; Kline, 2016). The CFA results indicated an acceptable fit to the data: *χ*^2^ = 384.390, *χ*^2^/df = 2.147 (<5.00), GFI = 0.895, IFI = 0.962 (>0.90), TLI = 0.955 (>0.90), CFI = 0.961 (>0.90), and SRMR = 0.035 (<0.05). Although the GFI value was marginally below the recommended threshold of 0.90, the remaining fit indices indicated acceptable model fit.

Following commonly used criteria for measurement model evaluation, standardized factor loadings above 0.60 were considered acceptable, CR values above 0.70 indicated satisfactory internal consistency, and AVE values above 0.50 indicated acceptable convergent validity (Fornell and Larcker, 1981; Hair et al., 2009). All standardized factor loadings were statistically significant and exceeded 0.60. CR values for all constructs were above 0.70. AVE values exceeded 0.50 for five of the six constructs. The AVE for perceived interaction was 0.490, which was slightly below the conventional threshold of 0.50 but very close to the recommended cutoff. In addition, the CR for perceived interaction was 0.742, exceeding the recommended threshold of 0.70. Its three standardized factor loadings were significant and above 0.60. Therefore, the convergent validity of perceived interaction was regarded as marginal but acceptable. This interpretation was also supported by the content coverage of the construct, as the three items captured theoretically relevant aspects of EMI interaction, including overall interaction, teacher feedback, and peer communication. Detailed results are presented in Table 1.

**Table 1:** Composite reliability and average variance extracted.

Variables	Items	Unstd.	S. E.	*Z*-value	*P*	Std.	SMC	CR	AVE
Perceived support	ps1	1				0.784	0.615	0.857	0.600
	ps2	0.830	0.063	13.204	***	0.754	0.569		
	ps3	0.798	0.057	14.003	***	0.803	0.645		
	ps4	0.737	0.056	13.200	***	0.754	0.569		
Perceived interaction	pi1	1				0.754	0.569	0.742	0.490
	pi2	0.750	0.088	8.544	***	0.696	0.484		
	pi3	0.809	0.096	8.468	***	0.646	0.417		
Learning enjoyment	le1	1				0.880	0.774	0.939	0.755
	le3	1.044	0.046	22.696	***	0.890	0.792		
	le2	1.113	0.051	21.831	***	0.873	0.762		
	le4	0.961	0.046	20.916	***	0.855	0.731		
	le5	0.981	0.048	20.529	***	0.846	0.716		
Learning satisfaction	sat1	1				0.867	0.752	0.926	0.715
	sat3	1.173	0.062	18.893	***	0.828	0.686		
	sat2	1.050	0.051	20.711	***	0.872	0.760		
	sat4	0.990	0.052	19.054	***	0.832	0.692		
	sat5	1.071	0.057	18.917	***	0.828	0.686		
Continuance intention	ci1	1				0.810	0.656	0.891	0.672
	ci3	0.980	0.059	16.651	***	0.845	0.714		
	ci2	0.963	0.058	16.738	***	0.849	0.721		
	ci4	0.992	0.067	14.891	***	0.772	0.596		
Cognitive load	cl2	1.190	0.069	17.145	***	0.875	0.766	0.928	0.720
	cl1	1				0.773	0.598		
	cl3	1.093	0.067	16.367	***	0.843	0.711		
	cl4	1.060	0.063	16.949	***	0.867	0.752		
	cl5	1.168	0.068	17.264	***	0.880	0.774		

*** indicates *p* < 0.001.

To further assess discriminant validity, the heterotrait–monotrait ratio of correlations (HTMT) was examined with bootstrapped confidence intervals. Most HTMT values were below the commonly used threshold of 0.90 (Henseler et al., 2015). The HTMT values for learning enjoyment–learning satisfaction and learning satisfaction–continuance intention were slightly above this threshold, reflecting the close theoretical relationships among these constructs. However, the 95% bootstrapped confidence intervals for these two pairs did not include 1.00. In particular, the HTMT value between learning satisfaction and continuance intention was 0.907, with a 95% confidence interval of [0.865, 0.943]. These results suggest that although the constructs were closely related, they were not empirically identical. Detailed results are presented in Table 2.

**Table 2:** HTMT values and bootstrapped confidence intervals.

Pair	HTMT	95% CI	Pair	HTMT	95% CI
(PS, PI)	0.771	[0.684, 0.853]	(PI, CI)	0.677	[0.563, 0.785]
(PS, LE)	0.578	[0.482, 0.665]	(LE, SAT)	0.907	[0.876, 0.936]
(PS, SAT)	0.625	[0.523, 0.721]	(LE, CL)	0.817	[0.769, 0.858]
(PS, CL)	0.556	[0.456, 0.645]	(LE, CI)	0.848	[0.804, 0.887]
(PS, CI)	0.571	[0.447, 0.685]	(SAT, CL)	0.782	[0.733, 0.827]
(PI, LE)	0.687	[0.596, 0.770]	(SAT, CI)	0.907	[0.865, 0.943]
(PI, SAT)	0.730	[0.627, 0.822]	(CL, CI)	0.703	[0.631, 0.765]
(PI, CL)	0.689	[0.603, 0.766]			

The chi-square difference tests further supported discriminant validity. Following established recommendations for covariance-based SEM, the constrained model in which the correlation between each pair of constructs was fixed to unity was compared with the unconstrained model (Anderson and Gerbing, 1988; Bagozzi et al., 1991). The chi-square differences were statistically significant in all cases, indicating that the constructs were empirically distinct. Detailed results are presented in Table 3. Taken together, the HTMT confidence-interval results and chi-square difference tests provided additional support for discriminant validity and an adequate basis for the subsequent structural model analysis.

**Table 3:** Discriminant validity of construct variables.

Unrestricted model *χ*^2^ = 575.371 (df = 284)	Restricted model *χ*^2^ (df = 285)	Δ*χ*^2^	Unrestricted model *χ*^2^ = 575.371 (df = 284)	Restricted model *χ*^2^ (df = 285)	Δ*χ*^2^
(PS, PI)	632.074	56.703***	(PI, CI)	657.614	82.243***
(PS, LE)	922.330	346.959***	(LE, SAT)	682.186	106.815***
(PS, SAT)	880.101	304.730***	(LE, CL)	896.305	320.934***
(PS, CL)	937.058	361.687***	(LE, CI)	705.300	129.929***
(PS, CI)	900.417	325.046***	(SAT, CL)	917.485	342.114***
(PI, LE)	667.034	91.663***	(SAT, CI)	614.978	39.607***
(PI, SAT)	649.622	74.251***	(CL, CI)	896.107	320.736***
(PI, CL)	667.727	92.356***			

*p-value < 0.05, **P-value < 0.01, ***P-value < 0.001.

### 4.2 Common method variance

Following common procedures for assessing common method bias in self-reported survey research, a single-factor CFA was estimated (Podsakoff et al., 2003). Using the same model fit criteria, the single-factor CFA showed poor model fit: *χ*^2^/df = 5.451, GFI = 0.708, IFI = 0.843, TLI = 0.824, CFI = 0.842, and SRMR = 0.079. These results indicated that a single common factor could not adequately account for the covariance among the measurement items.

In addition, several procedural remedies were implemented during data collection to mitigate potential method bias, including assurances of anonymity and the use of well-established measurement scales. Taken together, these statistical and procedural considerations suggested that common method bias is unlikely to have substantially affected the study’s findings.

### 4.3 Structural model assessment

The structural model demonstrated an acceptable fit to the data. Using the same model fit criteria, the model yielded *χ*^2^ = 392.176, *χ*^2^/df = 2.131 (< 5.00), GFI = 0.893, IFI = 0.961 (>0.90), TLI = 0.955 (>0.90), CFI = 0.961 (>0.90), and SRMR = 0.036 (<0.05). Although the GFI value was slightly below 0.90, the remaining fit indices indicated acceptable model fit. Overall, these results suggested that the proposed model provided an acceptable representation of the relationships among the six constructs in the EMI learning context.

### 4.4 Direct effects testing

The results showed that perceived support had a significant positive effect on learning satisfaction (*β* = 0.153, *p* < 0.001), supporting H1. Perceived interaction significantly predicted learning enjoyment (*β* = 0.720, *p* < 0.001), supporting H2. Learning enjoyment also had a significant positive effect on learning satisfaction (*β* = 0.830, *p* < 0.001), supporting H3. In addition, learning satisfaction showed a strong positive effect on continuance intention in EMI learning (*β* = 0.936, *p* < 0.001), supporting H4.

Although the standardized path coefficient between learning satisfaction and continuance intention was relatively high, the discriminant validity tests confirmed that these two variables were empirically distinct constructs. This strong association is consistent with expectation-confirmation logic, which positions satisfaction as a proximal antecedent of continuance intention. Detailed results are presented in Table 4.

**Table 4:** Path coefficients.

Path	Unstd.	S. E.	C. R.	*P*	Std.
Learning Satisfaction ← Perceived Support	0.201	0.052	3.881	***	0.153
Learning Enjoyment ← Perceived Interaction	1.010	0.093	10.823	***	0.720
Learning Satisfaction ← Learning Enjoyment	0.623	0.039	15.988	***	0.830
Continuance Intention ← Learning Satisfaction	1.133	0.072	15.671	***	0.936

*** indicates *p* < 0.001.

### 4.5 Mediation effects testing

The bootstrap results showed that all three indirect effects were statistically significant. Learning enjoyment significantly mediated the relationship between perceived interaction and learning satisfaction, supporting H5. Learning satisfaction significantly mediated the relationship between perceived support and continuance intention, supporting H6. Learning satisfaction also mediated the relationship between learning enjoyment and continuance intention, supporting H7. The confidence intervals for all indirect effects did not include zero. Detailed results are presented in Table 5. Taken together, these results indicated that learning satisfaction functioned as a key mediating variable linking learners’ learning experiences and emotional engagement with continuance intention in EMI learning.

**Table 5:** Mediation effects.

Mediation path	Point estimate	Product of coefficients		Bootstrapping (5000)			
				Bias-corrected 95%CI		Percentile 95% CI	
		SE	Z	Lower	Upper	Lower	Upper
Perceived Interaction → Learning Enjoyment (M) → Learning Satisfaction	0.630	0.071	8.873	0.504	0.782	0.502	0.779
Perceived Support → Learning Satisfaction (M) → Continuance Intention	0.228	0.076	3.000	0.068	0.372	0.068	0.374
Learning Enjoyment → Learning Satisfaction (M) → Continuance Intention	0.706	0.052	13.577	0.610	0.817	0.605	0.811

### 4.6 Moderation effect testing

To examine the moderating role of cognitive load proposed in H8, an interaction term was constructed from the mean-centered composite scores of learning enjoyment and cognitive load. The interaction term was then included in the structural model as an exogenous predictor of learning satisfaction.

**Figure 2:**
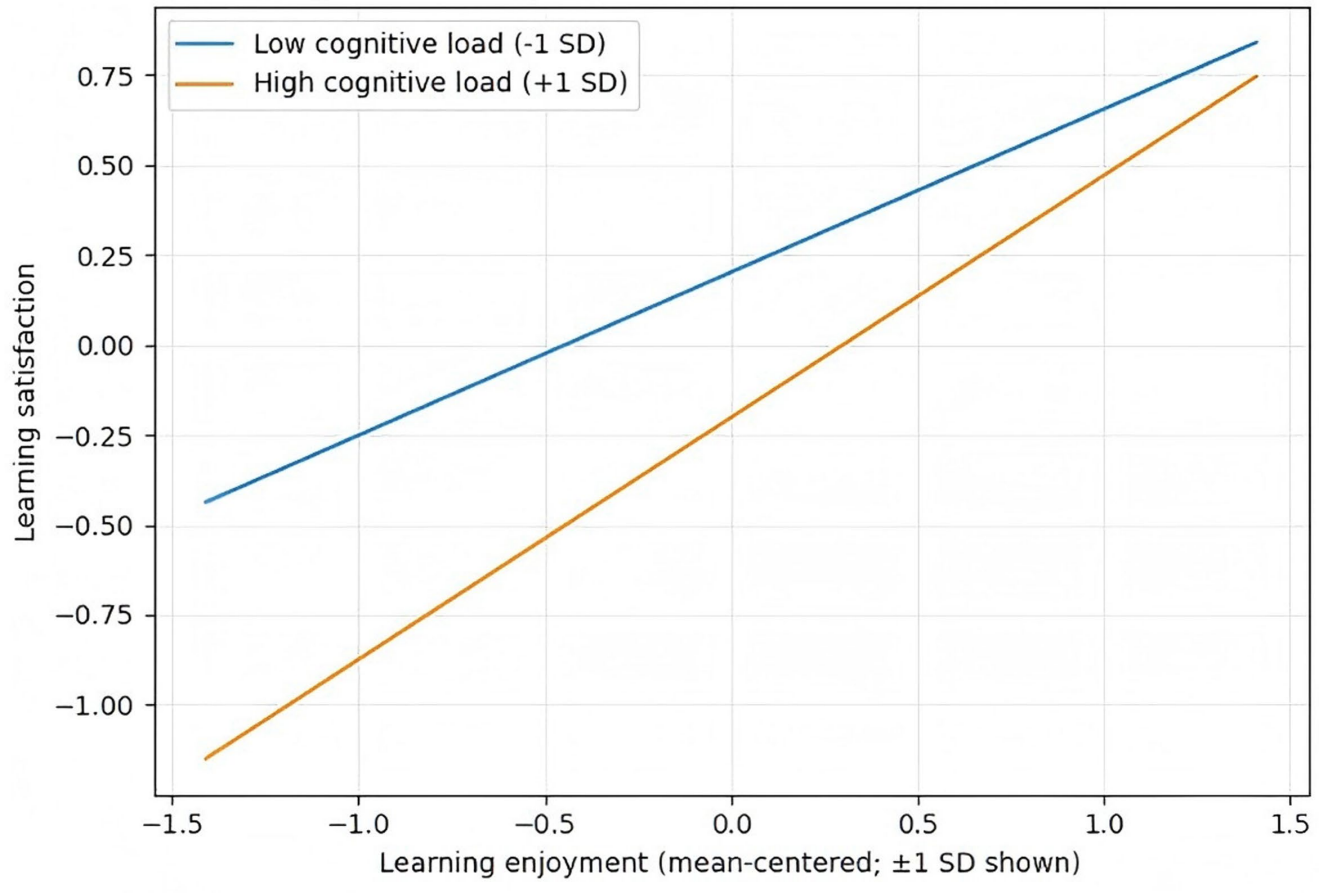
Moderating effect of cognitive load on the relationship between learning enjoyment and learning satisfaction in EMI learning. The plotted values represent predicted levels of learning satisfaction at low (−1 SD) and high (+1 SD) levels of cognitive load.

The results indicated a significant interaction effect between learning enjoyment and cognitive load on learning satisfaction (*p* < 0.001). Specifically, cognitive load positively moderated the relationship between learning enjoyment and learning satisfaction, such that the positive association between learning enjoyment and learning satisfaction was stronger at higher levels of cognitive load (see Figure 2). Accordingly, H8 was supported.

## 5 Discussion

### 5.1 Differentiated affective pathways of support and interaction

Perceived support and perceived interaction both emerged as important EMI learning experiences, but they did not operate in the same way. Perceived support was positively associated with learning satisfaction, whereas perceived interaction was positively associated with learning enjoyment. This distinction is important because support and interaction are often discussed together as favorable classroom conditions. In the present model, however, support was more closely tied to learners’ evaluation of EMI learning, while interaction was more closely tied to their positive emotional engagement.

The direct path from perceived support to learning satisfaction is understandable in the EMI context. EMI learners have to engage with disciplinary content through English, so learning difficulty often comes not only from the subject matter itself but also from the language through which it is accessed (Macaro et al., 2018; Pun and Jin, 2022). In this situation, clear guidance, timely help, and emotional or informational support can make challenging tasks feel more manageable. Support therefore seems to contribute to satisfaction by reducing uncertainty and helping learners judge the EMI experience as structured and worthwhile. This interpretation is consistent with expectation-confirmation logic, in which satisfaction reflects learners’ evaluation of whether their actual experiences meet or confirm prior expectations (Oliver, 1980; Bhattacherjee, 2001). It is also compatible with related online collaborative learning research showing that social support forms part of the conditions that shape students’ cognitive and evaluative experiences during learning (Cheng et al., 2023).

Perceived interaction worked through a different affective route. Its positive association with learning enjoyment suggests that interaction is closer to emotional activation than to overall evaluative judgment. In EMI classrooms, interaction gives students opportunities to ask questions, test their understanding, negotiate meaning, and receive responses from teachers or peers. These exchanges can make effortful EMI learning feel more participatory and socially meaningful. This interpretation is consistent with EMI research that views classroom interaction as important for meaning negotiation, communicative engagement, and positive learning experiences (Kırkgöz et al., 2023; Dai and Wang, 2024). In the present study, interaction appears to contribute first to enjoyment, which then contributes to satisfaction. Taken together, these results indicate that perceived support and perceived interaction contribute to EMI learners’ affective evaluation through different routes rather than operating as interchangeable classroom conditions.

### 5.2 Affective evaluation as the bridge from enjoyment to continuance

Positive emotional engagement became relevant to continuance intention mainly through learners’ satisfaction with EMI learning. Learning enjoyment was positively associated with learning satisfaction and mediated the path from perceived interaction to satisfaction. Satisfaction further mediated the paths from perceived support and learning enjoyment to continuance intention. These results suggest that learners do not continue EMI learning simply because the classroom is supportive or interactive, nor only because some activities are enjoyable. Such experiences matter when they are incorporated into a favorable evaluation of EMI learning.

Learning enjoyment made a clear contribution to satisfaction. This pattern fits achievement emotion research, where enjoyment is linked to learners’ sense of value, control, and engagement in learning (Pekrun, 2006; Pekrun, 2024). In EMI learning, enjoyment has a specific meaning because it often emerges while students are negotiating language and content at the same time. When learners enjoy EMI tasks despite difficulty, this emotion suggests that their effort is meaningful and that the activity remains within their reach. This helps explain why enjoyment contributed to satisfaction in the present model.

The mediation from interaction to satisfaction through enjoyment is also important. It suggests that interaction does not necessarily improve satisfaction by itself. In EMI classrooms, interaction may first make learning more emotionally rewarding: students can ask questions, test ideas, receive responses, and feel that they are participating rather than merely coping. EMI studies on classroom emotions and engagement offer a useful context for interpreting this result, as enjoyment has been linked to participation, willingness to communicate, and emotional experience in EMI-related learning contexts (Dai and Wang, 2024; Xu et al., 2025; Sun and Yang, 2025). Related online-learning evidence also suggests that the relationship between interaction and satisfaction may not be straightforward (Zhang et al., 2023). In the present study, interaction appears to become meaningful for learners by first supporting enjoyment, which then contributes to satisfaction.

Satisfaction then became the key bridge to continuance intention. The strong path from satisfaction to continuance intention is consistent with expectation-confirmation and continuance studies that place satisfaction close to continued use or continued engagement (Bhattacherjee, 2001; Ye et al., 2022; Zhang et al., 2023). In this study, satisfaction appears to summarize learners’ judgment of whether EMI learning is manageable, valuable, and worth sustaining. This is why both support and enjoyment affected continuance intention through satisfaction. Support can make the learning experience more reliable and manageable, while enjoyment can make the experience more rewarding; but the decision to continue depends on whether learners evaluate the overall experience positively. Taken together, these results indicate that positive emotional experience becomes relevant to continuance intention when it is integrated into learners’ broader satisfaction with EMI learning.

### 5.3 Cognitive load as a boundary condition for the enjoyment–satisfaction link

The moderation result adds a more conditional understanding of cognitive load in EMI learning. Cognitive load strengthened the relationship between learning enjoyment and learning satisfaction, indicating that enjoyment was more strongly associated with satisfaction when learners reported higher cognitive load. The issue is not whether cognitive load is beneficial in itself, but whether enjoyment experienced under higher cognitive demand carries stronger evaluative meaning.

This result needs to be interpreted against the demanding nature of EMI learning. EMI learners are required to engage with disciplinary content through English, so cognitive effort is built into the learning experience rather than added from the outside (Macaro et al., 2018; Pun and Jin, 2022). Prior EMI and related online-learning studies have tended to emphasize the potential costs of high cognitive demand, including increased processing difficulty in EMI learning and lower expectation confirmation, perceived usefulness, and satisfaction in online collaborative learning (Macaro et al., 2018; Pun and Jin, 2022; Cheng et al., 2023). Cognitive load theory also explains why this concern is reasonable: excessive processing demands may overload working memory and weaken learning outcomes (Sweller, 1988; Paas et al., 2003). The present result, however, shows that cognitive load can also change how enjoyment is evaluated in EMI learning.

In the present model, enjoyment mattered more for satisfaction when cognitive load was higher. A plausible explanation is that enjoyment experienced under demanding conditions carries greater evaluative weight. When EMI learning is relatively easy, enjoyment may mainly reflect a pleasant classroom experience. When EMI learning is demanding, however, enjoyment may signal that learners are coping with challenge, making progress, and finding value in effortful engagement. Under these conditions, enjoyment is not only a positive emotion; it also becomes evidence that a difficult learning experience remains manageable and worthwhile. This helps explain why the enjoyment–satisfaction link was stronger at higher levels of cognitive load.

This interpretation is compatible with recent cognitive-load research that treats cognitive load in relation to the source, complexity, and manageability of learning demands rather than as a single undifferentiated burden (Skulmowski and Xu, 2022; Zeitlhofer et al., 2024). In EMI learning, a certain level of cognitive demand may reflect active engagement with complex linguistic and disciplinary material. The key issue is therefore not whether cognitive load exists, but how learners experience and interpret the effort required by EMI learning. When learners can experience enjoyment while working through cognitive demand, that enjoyment becomes more closely tied to satisfaction.

Taken together, the moderation result refines the role of cognitive load in the overall model. Cognitive load did not act as a direct source of satisfaction; rather, it shaped the extent to which positive emotional experience was translated into evaluative judgment. In this sense, cognitive load functions as a boundary condition in the affective evaluation process. This helps explain why enjoyment may carry stronger evaluative meaning when EMI learning is experienced as cognitively demanding but still manageable.

## 6 Contributions and implications

### 6.1 Theoretical contributions

From a theoretical perspective, this study contributes to research on EMI learning and continuance intention in three ways. First, it extends expectation-confirmation logic to a learning context in which satisfaction is formed under sustained linguistic and cognitive demands. In conventional continuance models, satisfaction is placed close to continued use or continued engagement (Oliver, 1980; Bhattacherjee, 2001). In EMI learning, however, satisfaction has a more context-specific meaning. It reflects learners’ judgment that learning through English remains manageable and worthwhile even when disciplinary content has to be processed through a second language (Macaro et al., 2018; Pun and Jin, 2022). This positions satisfaction not simply as a general course-rating outcome, but as an affective evaluation mechanism linking EMI learning experience with continuance intention.

Second, the study clarifies the different roles of perceived support and perceived interaction in the affective pathway. Support and interaction are often discussed together as favorable classroom conditions, but the present findings suggest that they do not enter the model in the same way. Perceived support was directly associated with learning satisfaction, whereas perceived interaction was associated with learning enjoyment. This distinction helps explain why classroom conditions may not influence continuance intention through a single route. Their effects become clearer when they are understood as learning experiences that are first felt emotionally and then incorporated into learners’ evaluations of EMI learning.

Third, the study refines the role of cognitive load in EMI learning. Cognitive load theory explains why EMI may place pressure on learners’ working memory and attentional resources (Sweller, 1988; Paas et al., 2003). The present findings further suggest that cognitive load can also change the evaluative meaning of enjoyment. Enjoyment experienced under higher cognitive load appears to matter more for satisfaction, which is consistent with the view that cognitive load should be interpreted in relation to the source, complexity, and manageability of learning demands (Skulmowski and Xu, 2022; Zeitlhofer et al., 2024). In this sense, cognitive load is not treated simply as a barrier to EMI learning. Rather, it functions as a boundary condition that shapes when positive emotional experience becomes more strongly connected with learning satisfaction.

Together, these contributions support an affective-cognitive account of EMI continuance. This emphasis on an integrated affective-cognitive pathway is consistent with recent positive psychology research in language learning, which shows how cognitive appraisal, positive experiential states, and affective resources jointly shape learners’ emotional processes in foreign language learning (Jia et al., 2026). It is also broadly aligned with recent EFL writing research showing that learners’ mindsets, emotions, and self-regulated learning processes are closely interconnected rather than isolated factors (Sun et al., 2026). These related findings support the broader view that language learning persistence should be understood through the interaction of cognitive, affective, and regulatory processes. Support and interaction shape learners’ EMI experiences, enjoyment gives these experiences emotional value, satisfaction integrates them into broader evaluative judgments, and cognitive load conditions the strength of this process. Seen in this way, EMI continuance reflects learners’ judgment that a demanding learning experience is not only challenging, but also meaningful and worth sustaining.

### 6.2 Pedagogical implications

From a pedagogical perspective, these findings suggest that EMI course design should not be limited to adding support, increasing interaction, or reducing difficulty. The central issue is whether these conditions serve the affective functions identified in this study. Since perceived support was directly associated with learning satisfaction, support may be most useful when it helps learners view EMI learning as coherent, manageable, and worth sustaining. Related EFL research on perceived teacher support also suggests that students’ perceptions of support are context-sensitive and should not be treated as a uniform instructional condition (Wang et al., 2025). In EMI settings, this means that support may need to be aligned with learners’ specific linguistic, conceptual, and emotional difficulties. Rather than waiting until students encounter difficulty, teachers can make expectations, key disciplinary concepts, language demands, and task logic explicit before cognitively demanding work begins. In this way, support may reduce uncertainty before such uncertainty undermines satisfaction.

The association between perceived interaction and learning enjoyment points to a more selective use of classroom interaction. Interaction should not be evaluated simply by the amount of student talk, but by whether it creates moments of participation, response, and communicative success. EMI teachers can design tasks in which students use English to test disciplinary understanding, explain ideas to peers, compare interpretations, or solve content-based problems. Interaction becomes pedagogically valuable when it helps demanding EMI learning feel emotionally rewarding rather than merely linguistically demanding.

The mediating role of learning satisfaction suggests that support and enjoyment are relevant to continuance intention when learners integrate them into a broader judgment that EMI learning is valuable. EMI teachers may therefore need to make learners’ progress more visible, rather than focusing only on task completion. Staged outputs, brief reflective checkpoints, and comparisons between earlier and later understanding can help students recognize that their effort is producing academic growth. This is important because continuance intention depends less on isolated positive moments than on learners’ accumulated evaluation that EMI learning is worth continuing.

The moderating role of cognitive load also has a practical implication: EMI instruction should not aim simply to make learning easier. The results showed that enjoyment was more strongly related to satisfaction when cognitive load was higher, suggesting that enjoyment under challenge carries stronger evaluative value. Teachers therefore need to distinguish productive cognitive demand from unnecessary overload. Productive demand can be maintained through reasoning, application, comparison, and problem-solving tasks, whereas unnecessary overload can be reduced through clearer instructions, prior vocabulary and concept preparation, and gradual task sequencing. The goal is to keep EMI learning demanding enough to be meaningful, while giving learners enough structure to experience enjoyment as they work through challenge.

## 7 Conclusion

### 7.1 Summary of findings

This study examined the relationships between learners’ EMI experiences and their intention to continue EMI learning under cognitively demanding conditions. The results show that perceived support and perceived interaction contributed to different parts of the affective pathway. Perceived support was directly related to learning satisfaction, suggesting that support helps learners judge EMI learning as more manageable and worthwhile. Perceived interaction was related to learning enjoyment, indicating that interaction is more closely connected with learners’ positive emotional engagement in EMI learning.

Learning enjoyment also contributed to learning satisfaction and mediated the relationship between perceived interaction and learning satisfaction. Learning satisfaction, in turn, mediated the effects of perceived support and learning enjoyment on continuance intention. These findings indicate that continuance intention is shaped through learners’ broader evaluation of EMI learning, rather than by classroom conditions or positive emotion alone.

Cognitive load further shaped this pathway by strengthening the relationship between learning enjoyment and learning satisfaction. Enjoyment was more strongly associated with satisfaction when learners reported higher cognitive load, suggesting that enjoyment experienced under higher cognitive demand may carry stronger evaluative meaning. In sum, the findings show that EMI continuance is formed through the combined roles of support, interaction, enjoyment, satisfaction, and cognitive load.

### 7.2 Limitations and directions for future research

Several limitations should be considered when interpreting the findings. The study used cross-sectional self-report data, which limits the extent to which causal relationships can be inferred. Although the proposed model was theoretically grounded and supported by the SEM results, learners’ enjoyment, satisfaction, cognitive load, and continuance intention are likely to change over time. Future research could use longitudinal designs to examine how these affective and cognitive processes develop across different stages of EMI learning.

The sample was drawn from higher education institutions in China. This context is important for EMI research, but the findings may not fully represent EMI learning in other institutional, disciplinary, or cultural settings. Future studies could test the model in different countries, universities, academic disciplines, and course types to examine whether the affective-cognitive pathways identified here remain stable across contexts.

Cognitive load was also treated as a general perceived construct in this study. This was appropriate for examining its moderating role in the proposed model, but it does not show whether different types of cognitive load may function differently. Future research could distinguish intrinsic, extraneous, and germane cognitive load to examine when cognitive demand becomes productive and when it becomes overwhelming in EMI learning.

Finally, this study focused on learners’ perceptions and continuance intention rather than actual continued engagement or academic performance. Future research could combine survey data with behavioral indicators, classroom observations, interviews, or learning analytics. Such approaches would provide a fuller understanding of how EMI learners experience, evaluate, and sustain engagement in cognitively demanding learning environments.

## Data Availability

The raw data supporting the conclusions of this article will be made available by the authors, without undue reservation.
