# Supplementary material for: From support and interaction to continuance: affective pathways and the bright side of cognitive load in English-medium instruction
Source: Front Psychol. 2026 Jul 22;17:1806932. doi: 10.3389/fpsyg.2026.1806932 (PMC13438293; doi:10.3389/fpsyg.2026.1806932)
Supplement: Supplementary file 1 [file Table_1.DOCX]

**Supplementary Material S1: Measurement items, sources, and final questionnaire**

Table S1 reports the item codes, brief item content, and source scales for the six latent constructs examined in the study.

**Table S1. Measurement items and source scales**

| Construct | Item code | Brief item content | Source scale(s) |
| --- | --- | --- | --- |
| Perceived support | PS1 | Guidance on how to learn in EMI courses | Hu et al. (2023); Cheng et al. (2023) |
|  | PS2 | Emotional support when encountering EMI learning difficulties |  |
|  | PS3 | Information, advice, and guidance for improving EMI learning |  |
|  | PS4 | Practical help when facing EMI learning difficulties |  |
| Perceived interaction | PI1 | Effective interaction with teachers and classmates | Cheng et al. (2023); Pan et al. (2024) |
|  | PI2 | Timely teacher response and feedback |  |
|  | PI3 | Timely communication and discussion with classmates |  |
| Learning enjoyment | LE1 | Interest in EMI course learning | Alam et al. (2021) |
|  | LE2 | Preference for EMI courses |  |
|  | LE3 | Enjoyment of the EMI learning process |  |
|  | LE4 | Immersion in EMI course learning |  |
|  | LE5 | Sense of fulfillment from EMI course learning |  |
| Learning satisfaction | SAT1 | Satisfaction with the EMI learning process | Du et al. (2023); Pan et al. (2024) |
|  | SAT2 | Satisfaction with EMI learning outcomes |  |
|  | SAT3 | Pleasant overall EMI learning experience |  |
|  | SAT4 | Satisfaction with EMI learning results |  |
|  | SAT5 | Perceived wisdom of learning through EMI courses |  |
| Continuance intention | CI1 | Willingness to continue learning through EMI courses | Taghizadeh et al. (2021); Alam et al. (2021) |
|  | CI2 | Willingness to take EMI courses regularly in the future |  |
|  | CI3 | Willingness to increase EMI course learning frequency |  |
|  | CI4 | Preference for EMI courses over Chinese-taught courses |  |
| Cognitive load | CL1 | Frustration during EMI course learning | Cheng et al. (2023) |
|  | CL2 | Stress during EMI course learning |  |
|  | CL3 | Energy consumption during EMI course learning |  |
|  | CL4 | Complexity of EMI course learning |  |
|  | CL5 | Difficulty of EMI course learning |  |

Note: The full wording of all adapted items is presented in the final questionnaire below.

**Final questionnaire used in the study**

The original questionnaire was administered in Chinese. English translations are provided for reference. Unless otherwise stated, all construct items were measured on a seven-point Likert scale ranging from 1 = strongly disagree to 7 = strongly agree.

**全英文授课模式下大学生学习情况调查**

**Survey on University Students’ Learning Experiences in English-Medium Instruction Courses**

为深入了解全英文授课模式下大学生的学习情况，我们特开展此次调查。你的回答将帮助我们更好地理解学生的体验和需求，为高校教学改革提供重要参考。本问卷大约需要5-8分钟完成，所有回答将严格保密，仅用于学术研究。谢谢你的支持与配合！

This survey aims to understand university students’ learning experiences in English-medium instruction courses. Your responses will help us better understand students’ experiences and needs and provide useful evidence for teaching reform in higher education. The questionnaire takes approximately 5–8 minutes to complete. All responses will be kept confidential and used only for academic research. Thank you for your support and participation.

说明：全英文授课课程是指以英语为媒介教授的非语言学科课程，例如全英文教授的管理学、经济学等专业课程，不包括全英文教授的英语语音、英语视听说等语言类课程。

Note: English-medium instruction courses refer to non-language subject courses taught through English, such as management, economics, or other disciplinary courses delivered in English. They do not include language-skill courses taught in English, such as English pronunciation, English listening and speaking, or similar English-language courses.

Response scale for Q1–Q26:

1 = 非常不同意 / Strongly disagree
2 = 不同意 / Disagree
3 = 有点不同意 / Somewhat disagree
4 = 中立 / Neutral
5 = 有点同意 / Somewhat agree
6 = 同意 / Agree
7 = 非常同意 / Strongly agree

Q1 / PS1
在全英文授课课程学习中，老师和同学会很好地告诉我如何学习。
In EMI course learning, teachers and classmates clearly tell me how to learn.

Q2 / PS2
在全英文授课课程学习中遇到困难时，我能从老师和同学那里获得同情、关心等情感支持。
When I encounter difficulties in EMI course learning, I can receive emotional support, such as empathy and care, from teachers and classmates.

Q3 / PS3
在全英文授课课程学习中，老师和同学会提供相关的信息、建议和指导，帮助我提升学习效果。
In EMI course learning, teachers and classmates provide relevant information, advice, and guidance to help me improve my learning effectiveness.

Q4 / PS4
在全英文授课课程学习中遇到困难时，我可以向老师和同学寻求切实的帮助。
When I encounter difficulties in EMI course learning, I can seek practical help from teachers and classmates.

Q5 / PI1
在全英文授课课程学习中，我可以有效地与老师和同学互动。
In EMI course learning, I can interact effectively with teachers and classmates.

Q6 / PI2
在全英文授课课程学习中，老师能够及时对我的问题进行回答和反馈。
In EMI course learning, teachers can respond to my questions and provide feedback in a timely manner.

Q7 / PI3
在全英文授课课程学习中，我和其他同学可以及时沟通和讨论。
In EMI course learning, my classmates and I can communicate and discuss in a timely manner.

Q8 / LE1
全英文授课课程学习对我来说很有趣。
EMI course learning is interesting to me.

Q9 / LE2
全英文授课课程是我最喜欢的课程类型。
EMI courses are my favorite type of courses.

Q10 / LE3
我很享受全英文授课课程的学习过程。
I enjoy the learning process of EMI courses.

Q11 / LE4
在全英文授课课程学习中，我能沉浸其中。
I can become immersed in EMI course learning.

Q12 / LE5
在全英文授课课程学习中，我能获得满足感。
I can gain a sense of fulfillment from EMI course learning.

Q13 / SAT1
总体而言，我对全英文授课课程的学习过程是满意的。
Overall, I am satisfied with the learning process of EMI courses.

Q14 / SAT2
总体而言，我对全英文授课课程的学习效果是满意的。
Overall, I am satisfied with the learning outcomes of EMI courses.

Q15 / SAT3
总体而言，全英文授课课程的学习体验是令人愉快的。
Overall, the learning experience of EMI courses is pleasant.

Q16 / SAT4
总体而言，全英文授课课程的学习结果是令人满意的。
Overall, the learning results of EMI courses are satisfactory.

Q17 / SAT5
学习全英文授课课程是一个明智的决策。
Learning through EMI courses is a wise decision.

Q18 / CI1
基于当前的学习体验，我愿意继续学习全英文授课课程。
Based on my current learning experience, I am willing to continue learning through EMI courses.

Q19 / CI2
着眼未来发展，我愿意在将来定期研习全英文授课课程。
Considering my future development, I am willing to take EMI courses regularly in the future.

Q20 / CI3
我愿意增加全英文授课课程的学习频率。
I am willing to increase the frequency of learning EMI courses.

Q21 / CI4
相较于中文授课的常规课程，我更愿意学习全英文授课课程。
Compared with regular courses taught in Chinese, I am more willing to study EMI courses.

Q22 / CL1
全英文授课课程学习让我感到很沮丧。
EMI course learning makes me feel frustrated.

Q23 / CL2
全英文授课课程学习让我感到压力很大。
EMI course learning makes me feel very stressed.

Q24 / CL3
全英文授课课程学习耗费我太多精力。
EMI course learning consumes too much of my energy.

Q25 / CL4
全英文授课课程学习太复杂。
EMI course learning is too complex.

Q26 / CL5
全英文授课课程学习非常困难。
EMI course learning is very difficult.

Demographic information 背景信息

Q27
你的性别是？
What is your gender?

Response options:
男性 / Male
女性 / Female

Q28
你的年级是？
What is your academic year?

Response options:
大学一年级 / First year
大学二年级 / Second year
大学三年级 / Third year
大学四年级 / Fourth year
